# A Qualitative Comparison of the Abbott SARS-CoV-2 IgG II Quant Assay against Commonly Used Canadian SARS-CoV-2 Enzyme Immunoassays in Blood Donor Retention Specimens, April 2020 to March 2021

**DOI:** 10.1128/spectrum.01134-22

**Published:** 2022-06-02

**Authors:** Kento T. Abe, Bhavisha Rathod, Karen Colwill, Anne-Claude Gingras, Ashleigh Tuite, Ninette F. Robbins, Guillermo Orjuela, Craig Jenkins, Valerie Conrod, Qi-Long Yi, Sheila F. O’Brien, Steven J. Drews

**Affiliations:** a Lunenfeld-Tanenbaum Research Institute at Mt. Sinai Hospital, Sinai Health, Toronto, Ontario, Canada; b Department of Molecular Genetics, University of Toronto, Toronto, Ontario, Canada; c Treadwell Therapeutics, Toronto, Ontario, Canada; d Dalla Lana School of Public Health, University of Toronto, Toronto, Ontario, Canada; e Scientific Affairs, Abbott Transfusion Medicine, Chicago, Illinois, USA; f Scientific Affairs, Abbott Transfusion Medicine, Bogotá, Colombia; g COVID-19 Serological Screening Laboratory, Canadian Blood Services, Ottawa, Ontario, Canada; h Epidemiology and Surveillance, Canadian Blood Services, Ottawa, Ontario, Canada; i School of Epidemiology and Public Health, University of Ottawa, Ottawa, Ontario, Canada; j Canadian Blood Services, Microbiology, Edmonton, Alberta, Canada; k Department of Laboratory Medicine and Pathology, University of Alberta, Edmonton, Alberta, Canada; Johns Hopkins Hospital

**Keywords:** IgG, SARS-CoV-2 antibody, methods comparisons, nucleocapsid, receptor binding domain, spike

## Abstract

Our group has previously used laboratory and commercially developed assays to understand the IgG responses to SARS-CoV-2 antigens, including nucleocapsid (N), spike (S), and receptor binding domain (RBD), in Canadian blood donors. In this current study, we analyzed 17,428 available and previously characterized retention samples collected from April 2020 to March 2021. The analysis compared the characteristics of the Abbott SARS-CoV-2 IgG II Quant assay (Abbott anti-spike [S], Abbott, Chicago, IL) against four other IgG assays. The Abbott anti-S assay has a qualitative threshold of 50 AU/mL. The four comparator assays were the Abbott anti-nucleocapsid (N) assay and three commonly used Canadian in-house IgG enzyme-linked immunosorbent assays (ELISAs) recognizing distinct recombinant viral antigens, full-length spike glycoprotein, glycoprotein RBD, and nucleocapsid. The strongest qualitative relationship was between Sinai RBD and the Abbott anti-S assay (kappa, 0.707; standard error [SE] of kappa, 0.018; 95% confidence interval, 0.671 to 0.743). We then scored each previously characterized specimen as positive when two anti-SARS-COV-2 assays identified anti-SARS-CoV-2 IgG in the specimen. Using this composite reference standard approach, the sensitivity of the Abbott anti-S assay was 95.96% (95% confidence interval [CI], 93.27 to 97.63%). The specificity of the Abbott anti-S assay was 99.35% (95% CI, 99.21 to 99.46%). Our study provides context on the use of commonly used SARS-CoV-2 serologies in Canada and identifies how these assays qualitatively compare to newer commercial assays. Our next steps are to assess how well the Abbott anti-S assays quantitatively detect wild-type and SARS-CoV-2 variants of concern.

**IMPORTANCE** We describe the qualitative test characteristics of the Abbott SARS-CoV-2 IgG II Quant assay against four other anti-SARS-CoV-2 IgG assays commonly used in Canada. Although there is no gold standard for identifying anti-SARS-CoV-2 seropositivity, aggregate standards can be used to assess seropositivity. In this study, we used a specimen bank of previously well-characterized specimens collected between April 2020 and March 2021. The Abbott anti-S assay showed the strongest qualitative relationship with a widely used laboratory-developed IgG assay for the SARS-CoV-2 receptor binding domain. Using the composite reference standard approach, we also showed that the Abbott anti-S assay was highly sensitive and specific. As new anti-SARS-CoV-2 assays are developed, it is important to compare their test characteristics against other assays that have been extensively used in prior research.

## INTRODUCTION

Canadian Blood Services previously engaged a broad group of laboratories in North America to attempt to understand the neutralizing capacity of blood donor antibodies to SARS-CoV-2 (1–5). Originally, much of this preliminary work was focused on supporting SARS-CoV-2 convalescent plasma studies in Canada (1, 2, 4, 5). The identification of waning neutralizing antibody responses in blood donors (1) led to the development of a further “Correlates of Immunity” project, which had the stated goal of understanding changes in anti-SARS-CoV-2-neutralizing capacity as the COVID-19 pandemic advanced. As part of this Correlates of Immunity project, our group was able to sample 1,500 retention specimens a month from Canadian blood donors using a repeated cross-sectional design with random cross-sectional sampling of all available retention samples for a 12-month period from April 2020 until March 2021 (6–10). During this process, we used a variety of assays that have been widely used by our and other groups to assess SARS-CoV-2 seroprevalence in Canada. These included the Abbott Architect antinucleocapsid antigen IgG assay (Abbott-NP, Abbott, Chicago, IL), as well as three in-house Sinai Health (Toronto, ON, Canada) IgG enzyme-linked immunosorbent assays (ELISAs) utilizing recombinant viral antigens, full-length spike glycoprotein (S), spike glycoprotein receptor binding domain (RBD), and nucleocapsid (NP) (2, 3, 6, 11–16). The Sinai Health IgG ELISAs were developed to allow for the scalable parallel detection of IgGs against the S, RBD, and NP. They are described in extensive detail in the literature (12). Apart from work undertaken with Canadian Blood Service, the laboratory-developed automated ELISAs described are being used or have been used in multiple Canadian studies, including the Canadian COVID-19 Antibody and Health Survey from Statistics Canada (17), the Action to Beat Coronavirus study (18), and studies focused on infection and/or vaccine responses across different cohorts predicted to have a weaker immune response (19, 20).

Given the absence of a gold standard (6, 21), we previously characterized SARS-CoV-2 seropositivity, including latent class analysis (7, 8) and composite reference standard approaches (6). We also attempted to understand the impact of donor-declared vaccine history on SARS-CoV-2 serological profiles (1, 2, 9, 10). We have noted that responses of different assays are, at times, inconsistent with one another (1, 6–9). These differences may be due to a variety of factors, including COVID-19 vaccination, false-positive results (probably less likely), assay accuracy and reliability, cross-reactivity with seasonal coronaviruses, and antibody waning for both anti-N and anti-S targets (6–8, 22). In our prior analysis of specimens from the Correlates of Immunity project, we were able to determine that regardless of the approach, by March of 2021, infection and vaccine-mediated seroprevalence in Canadian blood donors (<10%) was much lower than U.S. seroprevalence estimates. We also noted that our specimen bank was influenced by relatively low infection rates and the relatively slow ramp-up of vaccination programs (6, 7, 23, 24).

The development of new anti-SARS-CoV-2 commercial assays allows for the operationalization of seroprevalence studies by non-research-focused laboratories, including clinical and public health laboratories as well as blood operators (7, 21, 25, 26). The Abbott SARS-CoV-2 Quant assay (Abbott, Chicago, IL) was developed for the qualitative and quantitative determination of IgG against SARS-CoV-2 S. The qualitative cutoff for this assay has been described as 50 AU/mL (27). This assay has now been used in multiple seroprevalence surveys (28–31). Due to the nonavailability of some retention specimens from our specimen bank, this study did not attempt to infer neutralizing antibody seroprotection from the seroprevalence estimates. Instead, we attempted to understand qualitatively the test characteristics of the Abbott anti-S assay against specimens well characterized by qualitative assays previously utilized by Canadian seroprevalence and health studies.

## RESULTS

### Study population characteristics.

Retention specimens from a total of 17,428 blood donors were included in the study, with samples collected between April 2020 and March 2021. Epidemiological characterization of these blood donors was previously described in multiple publications (6, 7).

### Percentage agreement between the Abbott anti-S assays and Abbott anti-N, Sinai anti-S, Sinai anti-RBD, and Sinai anti-N assays.

The percentage agreement estimates between the Abbott anti-S assay and Abbott anti-N, Sinai anti-S, Sinai anti-RBD, and Sinai anti-N assays are listed in Tables 1 to 4. The highest agreement between positive Abbott anti-S assay results was with positive Sinai anti-S results (72.6%; Table 2), then positive Sinai anti-RBD results (66.6%; Table 3), then positive Sinai anti-N results (32.3%; Table 4), and, finally, positive Abbott anti-N results (28.7%; Table 1). The highest agreement between negative Abbott anti-S results was with negative Sinai anti-RBD results (99.5%; Table 3), then negative Sinai anti-N results (97.7%), as well as negative Abbott anti-N results (99.7%; Table 1), and, finally, negative Sinai anti-N results (97.4%; Table 2).

**TABLE 1:** Comparison of Abbott-anti-S and Abbott anti-N assays

Abbott anti-S result	No. (%)^*a*^ of results		Total
	Abbott anti-N positive	Abbott anti-N negative	
Positive	134 (28.7)	333	467
Negative	43	16,918 (99.7)	16,961
Total	177	17,251	17,428

a Numbers in parentheses represent percent agreement versus other methodology.

**TABLE 2:** Comparison of Abbott-anti-S and Sinai anti-S assays

Abbott anti-S result	No. (%)^*a*^ of results		Total
	Sinai anti-S positive	Sinai anti-S negative	
Positive	339 (72.6)	128	467
Negative	443	16,518 (97.4)	16,961
Total	782	16,646	17,428

a Numbers in parentheses represent percent agreement versus other methodology.

**TABLE 3:** Comparison of Abbott anti-S and Sinai anti-RBD assays

Abbott anti-S result	No. (%)^*a*^ of results		Total
	Sinai anti-RBD positive	Sinai anti-RBD negative	
Positive	311 (66.6)	156	467
Negative	93	16,868 (99.5)	16,961
Total	404	17,024	17,428

a Numbers in parentheses represent percent agreement versus other methodology.

**TABLE 4:** Comparison of Abbott-anti-S and Sinai anti-N assays

Abbott anti-S result	No. (%)^*a*^ of results		Total
	Sinai anti-N positive	Sinai anti-N negative	
Positive	151 (32.3)	316	467
Negative	392	16,569 (97.7)	16,961
Total	543	16,885	17,428

a Numbers in parentheses represent percent agreement versus other methodology.

### Comparison of agreement between qualitative results (kappa analysis).

Qualitative determination of positive results used signal-to-cutoff values, which are described in the Materials and Methods. The distribution of qualitative agreement between the Abbott anti-S assays and Abbott anti-N (Table 1), Sinai anti-S (Table 2), Sinai anti-RBD (Table 3), and Sinai anti-N (Table 4) were determined. The highest kappa was with Sinai anti-RBD (kappa, 0.707; SE of kappa, 0.018; 95% confidence interval (CI), 0.671 to 0.743) and progressively lower for Sinai anti-S (kappa, 0.527; SE of kappa, 0.020; 95% CI, 0.489 to 0.565), Abbott anti-N (kappa, 0.407; SE of kappa, 0.030; 95% CI, 0.348 to 0.467), and lowest for Sinai anti-N (kappa, 0.278; SE of kappa, 0.027; 95% CI, 0.226 to 0.3330).

### Analysis of discordant specimens positive by Abbott anti-S.

**FIG 1:**
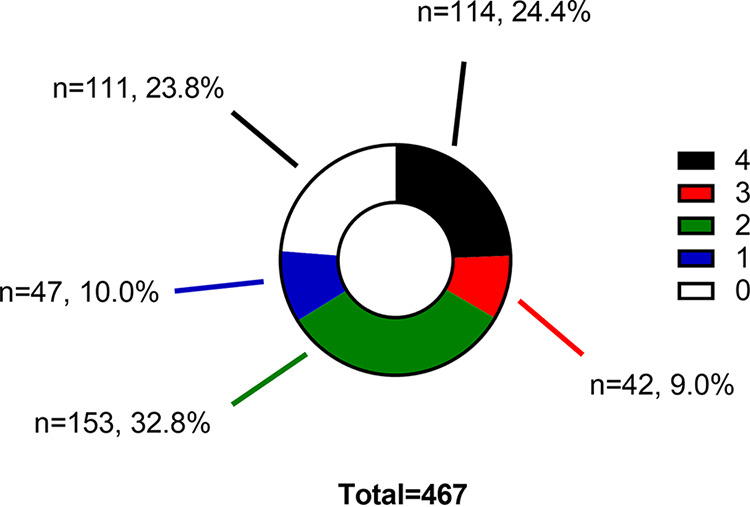
Reactivity of Abbott anti-S-positive specimens with other anti-SARS-CoV-2 IgG assays. The graph indicates the percentage and number of Abbott-anti-S-positive specimens that were reactive (1 to 4) and nonreactive by other anti-SARS-CoV-2 IgG assays.

**FIG 2:**
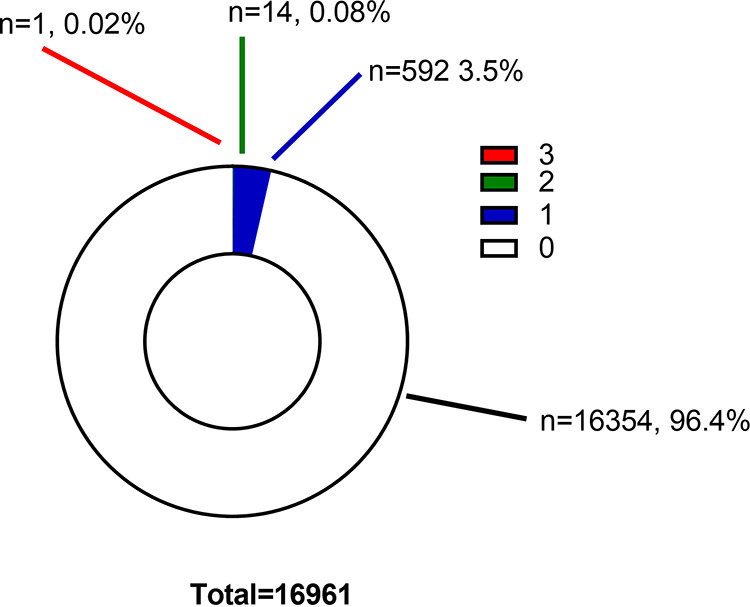
Reactivity of Abbott anti-S-negative specimens with other anti-SARS-CoV-2 IgG assays. The graph indicates the percentage and number of Abbott-anti-S-negative specimens that were reactive (1 to 3) and nonreactive by other anti-SARS-CoV-2 IgG assays.

Of the 467 specimens determined to be positive by the Abbott anti-S qualitative cutoff, distributions of positivity by other assays are identified in Fig. 1 and 2. Discordant specimens positive by Abbott anti-S and negative by all other assays or positive by only one other assay were analyzed as follows.

About a quarter of Abbott anti-S-positive specimens were negative on all other assays (i.e., their signal-to-cutoff values were below cutoff) (Fig. 1). None of these 111 specimens that were only Abbott anti-S positive had a sequentially prior Abbott anti-N-positive specimen (based on Canadian Institutes of Health Research [CIHR] number). None of the 111 specimens were from donors who declared a recent history of COVID-19 vaccination. Not only were target signals below cutoff, but in general, median signal-to-cutoff values were well below cutoff. Summary results for each of the targets suggested low median signal-to-cutoff values in this group of specimens as follows: Abbott anti-N (median, 0.02 [25th to 75 percentiles, 0.02 to 0.05]), Sinai anti-S (median, 0.08 [25th to 75 percentiles, 0.03 to 0.09]), Sinai anti-RBD (median, 0.06 [25th to 75 percentiles, 0.03 to 0.09]), Sinai anti-N (median, 0.06 [25th to 75 percentiles, 0.04 to 0.09]), and Abbott anti-S (median, 78.7 [25th to 75 percentiles, 57.5 to 112.2]).

Of the 467 specimens determined to be positive by the Abbott anti-S qualitative cutoff, about three-quarters were positive on at least one other assay. There were 47 determined distributions of positivity by only one other assay as in Fig. 1. The median Abbott anti-S signal-to-cutoff value for these 47 specimens was relatively low (median, 104.8 AU/mL 25th to 75 percentiles, 72.8 to 117.0 AU/mL). Distributions of other positive assays were Sinai anti-S (*n* = 30), Sinai anti-RBD (*n* = 10), Abbott anti-N (*n* = 4), and Sinai anti-N (*n* = 3). Of the specimens with lone positive assays, the median signal-to-cutoff ratios for positive specimens were generally low for most markers as follows: Abbott anti-N (median, 3.41 [25th to 75 percentiles, 2.09 to 5.05]), Sinai anti-S (median, 0.40 [25th to 75 percentiles, 0.29 to 0.57]), and Sinai anti-RBD (median, 0.23 25th to 75 percentiles, 0.20 to 0.27). Of note, these specimens may have a relatively strong Sinai anti-N (median, 3.34 25th to 75 percentiles, 0.45 to 3.67) signal-to-cutoff values.

### Analysis of discordant specimens negative by Abbott anti-S.

Of the 16,961 specimens determined to be negative by the Abbott anti-S qualitative assay, distributions of positivity by other assays are identified in Fig. 2.

As in Fig. 2, 15 Abbott anti-S negative specimens were determined to be positive by two (*n* = 14, 0.08%) or three (*n* = 1, 0.02%) other tests. Positive values are highlighted in Table 5. Frequencies of positivity were Abbott anti-N (*n* = 2; median, 2.21 [25th to 75 percentiles, 2.05 to 2.37]), Sinai anti-S (*n* = 14; median, 0.32 [25th to 75 percentiles, 0.29 to 0.55]), Sinai anti-RBD (*n* = 14; median, 0.23 [25th to 75 percentiles, 0.22 to 0,44]), and Sinai anti-N (*n* = 9; median, 0.58 [25th to 75 percentiles, 0.53 to 0.72]). Of the 15 specimens, the median signal to cutoff for the Abbott anti-S was relatively low (median, 0.8 [25th to 75 percentiles, 95% CI, 0.2 to 3.2]). In this group, the Abbott anti-S values for two specimens, CIHR013582 (46.8 AU/mL) and CIHR013757 (16.3 AU/mL), were below the qualitative cutoff of 50 AU/mL but above the quantitative reportable limit of detection (6.8 AU/mL) for the Abbott anti-S assay. One specimen (CIHR006065) had positive signals for Sinai anti-S (1.31), Sinai anti-RBD (0.49), and Sinai anti-N (1.92).

**TABLE 5:** Signal-to-cutoff ratios of four assays on which Abbott anti-S negative specimens were positive by two or more anti-SARS-CoV-2 assays

CIHR no.	Signal-to-cutoff ratio of:				Abbott anti-S negative specimens (AU/mL)
	Abbott anti-N	Sinai anti-S	Sinai anti-RBD	Sinai anti-N	
CIHR000106	0.08	0.31	0.22	0.08	3.4
CIHR002075	0.05	1.00	0.43	0.63	0
CIHR003710	0.04	0.26	0.57	0.51	0.7
CIHR006065	0.02	1.31	0.49	1.92	0
CIHR007833	0.23	0.89	0.20	0.81	0.8
CIHR008235	2.05	0.29	0.06	0.23	0.2
CIHR009609	0.45	0.29	0.30	0.25	1.8
CIHR012266	0.02	0.32	0.24	0.16	3.2
CIHR013582	0.02	0.44	0.60	0.02	46.8
CIHR013757	0.06	0.33	0.40	0.55	16.3
CIHR014210	2.37	0.12	0.22	0.58	0
CIHR015196	0.06	0.31	0.22	0.56	0.8
CIHR015226	0.02	0.38	0.19	0.05	1.2
CIHR015837	0.01	0.30	0.23	0.61	0.8
CIHR016791	0.08	0.20	0.22	0.45	1.2

### Sensitivity and specificity calculations.

Using a composite reference standard approach, the sensitivity of the Abbott anti-S assay was 95.96% (95% CI, 93.27 to 97.64%). The specificity of the Abbott anti-S assay was 99.35% (95% CI, 99.21 to 99.46%) (Table 6).

**TABLE 6:** Sensitivity and specificity calculation matrix using reference standards

Abbott anti-S result	No. of positive specimens (≥2 positive tests)	No. of negative specimens (<2 positive tests)	Total
Abbott anti-S positive	356	111	467
Abbott anti-S negative	15	16,946	16,961
Total	371	17,057	17,428

## DISCUSSION

This study compared the characteristics of the Abbott SARS-CoV-2 IgG II Quant assay (Abbott anti-spike [S]; Abbott, Chicago IL) against four other SARS-CoV-2 IgG assays that are commonly used in Canada (2, 3, 6, 11–16). This study did not attempt to infer neutralizing antibody seroprotection from the seroprevalence estimates and did not assess seroprevalence in Canadian blood donors.

The Abbott anti-S assay can be utilized as a qualitative (27, 32), as well as quantitative, assay (33–36) for the detection of anti-SARS-CoV-2 antibodies. The assay is a chemiluminescent microparticle immunoassay for IgG against the RBD region of S (21) with a qualitative assay cutoff of 50 AU/mL (27, 32). In the past, we have also utilized serological assays against N, S, and RBD, including those included in this study, to estimate seroprevalence in Canadian blood donors (6–8). We also noted that well-validated anti-S and anti-RBD assays allow for estimates of seroprevalence that are less impacted by waning antibodies as those seen with anti-N antibodies (6–8, 25, 37). The reasons for waning antibodies to both N and S antigens are still not completely understood but may reflect low levels of infections in some populations, waning immunity after vaccination, immune status in populations studies, and differences in vaccine rollout strategies (38–41). It is also important to utilize a variety of assays for seroprevalence work, given the absence of a gold-standard SARS-CoV-2 immunoassay (6).

Assays that bridge between qualitative binding and quantitative neutralization are important, and this study focused on the qualitative binding element of immunity. In our previous studies, we have shown a difference in detection of binding versus neutralizing anti-SARS-CoV-2 antibodies in specimens from the same cohort of blood donors in our Correlates of Immunity project (9, 10). Other work has shown that not all binding antibodies correlate to neutralization but that IgG against RBD (and to a lesser extent, S) can act as an indicator of neutralization (2, 42). In other cases, detection of both binding and neutralizing antibodies may both indicate immune protection after vaccination in macaque infection models (43). However, even some anti-RBD antibodies may bind to nonneutralizing faces of the RBD molecule (44). We now understand that antibodies to SARS-CoV-2 are polyfunctional and undertake neutralizing and antibody-dependent cell-mediated cytotoxicity (ADCC) and antibody-dependent cellular phagocytosis (ADCP) through antibodies to both N and S proteins (4, 45–47). However, the mechanisms of ADCC and ADCP are still being understood and require further studies in humans (48, 49).

The characterization of commercially available anti-S quantitative assays, such as the Abbott anti-S assay, is important for supporting large-scale serosurveys and for guiding public health decision-making (25, 37, 50). This ability of the Abbott serology platforms to test both anti-S and anti-N signals will play an important role in helping serosurveillance groups to characterize population-level immune responses to both vaccination and natural SARS-CoV-2 infection (29–31, 51, 52).

We acknowledge several important caveats in this study, including the use of a relatively small number of specimens over a 12-month period from April 2020 to March 2021. Because these were healthy blood donors, these donors did not provide clinical information on COVID-19 disease. The methodologies used to detect antibodies were qualitative or, in the case of the Abbott anti-S assay, were analyzed as qualitative assays and did not assess antibody titers over time. For a small subset of data, we assessed donor-declared vaccine histories and did not access health databases in jurisdictions where donors lived. In this study, we also did not account for the impact of variants of concern on how SARS-CoV-2 immunoassays are qualified and characterized (25, 53).

In conclusion, we describe the qualitative characteristics of the Abbott anti-S assay. We used a composite reference standard approach to estimate the sensitivity of the Abbott anti-S assay to be 95.96% (95% CI, 93.27 to 97.63%). We also estimated the specificity as 99.35% (95% CI, 99.21 to 99.46%). Our study provides context on the use of commonly used SARS-CoV-2 serologies in Canada and identifies how these assays qualitatively compare to newer commercial assays. Our next steps are to assess how well the Abbott anti-S assays quantitatively detect SARS-CoV-2 wild type and variants of concern.

## MATERIALS AND METHODS

### Ethical considerations.

This project received ethics board clearance from the following institutions: Canadian Blood Services, the University of Alberta, and Sinai Health, Toronto (Mount Sinai Hospital).

### CIHR Correlates of Immunity study participants and samples.

Canadian Blood Services has blood collection sites concentrated in large and small cities in all Canadian provinces except Quebec. Blood donors must meet the following criteria: be at least 17 years of age, pass health selection criteria screening, and pass infectious disease screening protocols for blood donations that are then used to manufacture products for transfusion. At each donation, there is also an additional EDTA plasma (Becton Dickson [BD], Mississauga, ON, Canada) retention sample collected for additional blood testing if required (54).

### Study design and population.

We designed a repeated cross-sectional design with a random cross-sectional sampling of all available retention samples (*n* = 1,500/month) for a 12-month period from April 2020 until March 2021. A two-stage process sampling approach was used with a random selection of blood donor clinics followed by a random sample selection within clinics. Samples were anonymized. We collected variables, including sex, birth year, residential forward sortation area (FSA; first three characters of postal code), donation date, and collection site, which were extracted from the Canadian Blood Services donor database. Retention plasma specimens were aliquoted at Canadian Blood Services and transported to test sites (6). A residual specimen was stored at −80°C for the remainder of the study.

### SARS-CoV-2 antibody testing.

Each retained plasma sample was evaluated for SARS-CoV-2 IgG antibodies using four assays as described previously (6). This study undertook parallel testing for the Abbott Architect anti-nucleocapsid antigen assay (Abbott-NP, Abbott, Chicago, IL), as well as three in-house IgG ELISAs utilizing recombinant viral antigens, full-length spike glycoprotein (spike), spike glycoprotein receptor binding domain (RBD), and nucleocapsid (NP) (11, 12). The testing of specimens by the Abbott-NP assay occurred in a sequential and ascending manner based on specimen number (CIHR number). Residual specimens that were previously tested by the Abbott-NP assay were available for testing by the Abbott SARS-CoV-2 Quant assay. Specimens used for this study included those described in a prior analysis for April 2020 to March 2021, inclusive (6).

### Thresholds used for assay comparisons.

Ratio-converted ELISA reads were undertaken as previously described (2, 11), and cutoffs (positive) for each of the targets were N, ≥0.396; RBD, ≥0.186; and S, ≥0.190 (6). Plasma samples were also tested with the Abbott Architect SARS-CoV-2 IgG test (Abbott Laboratories, USA), which detects anti-N IgG antibodies as directed by the manufacturer, using an antibody index (AI) cutoff of 1.4. Plasma samples were also tested with the Abbott Architect anti-S SARS-CoV-2 IgG test (Abbott Laboratories, USA), which detects the anti-S total with the cutoff of 50 AU/mL.

### Data storage and statistical analysis.

We used a Microsoft Excel (Redmond, WA, USA) spreadsheet for data storage. Data were analyzed as described in the results section using GraphPad Prism (v9.2.0; GraphPad Software, Inc., San Diego, CA, USA). The percentage agreement between the Abbott SARS-CoV-2 Quant assay and other methods was calculated based on the denominators of rows as per Tables 1 to 4 and Table 6. Kappa analysis was performed using GraphPad Prism Quick Calcs and interpretations as previously described (55, 56). Sensitivity and specificity calculations were undertaken using the Vassar Stats clinical calculator 1 with 95% confidence intervals estimated as per Cohen (57, 58).

### Collection of SARS-CoV-2 vaccination history in donors.

All donors at the time of donation were asked if they received a SARS-CoV-2 vaccine in the past 3 months. This was standard practice by Canadian Blood Services. Information on dosing and vaccine type was limited. Provincial vaccine databases are not linked to the blood operator records of donation.

### Determination of anti-SARS-CoV-2-positive and -negative specimens for analysis of sensitivity and specificity.

In the past, we have estimated positives in the sample set using multiple methods, including a composite reference standard where seropositivity using two or more assays described below represents a true-positive case (6). For this study, we continued to use that reference standard. Specimens were deemed to be anti-SARS-CoV-2 positive if they reacted with any two of the following previously characterized assays; Abbott anti-N, Sinai anti-S, Sinai anti-RBD, and Sinai anti-N. Negative specimens were reactive to only one of the assays listed above or none of these assays.

## Supplementary Material

Reviewer comments

## References

[1] Drews SJ, Devine DV, McManus J, Mendoza E, Manguiat K, Wood H, Girardin R, Dupuis A, McDonough K, Drebot M. 2021. A trend of dropping anti-SARS-CoV-2 plaque reduction neutralization test titers over time in Canadian convalescent plasma donors. Transfusion 61:1440–1446. doi:10.1111/trf.16364. 33734448

[2] Abe KT, Li Z, Samson R, Samavarchi-Tehrani P, Valcourt EJ, Wood H, Budylowski P, Dupuis AP, Girardin RC, Rathod B, Wang JH, Barrios-Rodiles M, Colwill K, McGeer AJ, Mubareka S, Gommerman JL, Durocher Y, Ostrowski M, McDonough KA, Drebot MA, Drews SJ, Rini JM, Gingras A-C. 2020. A simple protein-based surrogate neutralization assay for SARS-CoV-2. JCI Insight 5:e142362. doi:10.1172/jci.insight.142362. 32870820 PMC7566699

[3] Sheffield WP, Bhakta V, Howell A, Jenkins C, Serrano K, Johnson N, Lin Y‐CJ, Colwill K, Rathod B, Greenberg B, Gingras A‐C, Evans DH, Flaumenhaft E, Beckett A, Drews SJ, Devine DV. 2022. Retention of hemostatic and immunological properties of frozen plasma and COVID-19 convalescent apheresis fresh-frozen plasma produced and freeze-dried in Canada. Transfusion 62:418–428. doi:10.1111/trf.16772. 34907536

[4] Bégin P, Callum J, Jamula E, Cook R, Heddle NM, Tinmouth A, Zeller MP, Beaudoin-Bussières G, Amorim L, Bazin R, Loftsgard KC, Carl R, Chassé M, Cushing MM, Daneman N, Devine DV, Dumaresq J, Fergusson DA, Gabe C, Glesby MJ, Li N, Liu Y, McGeer A, Robitaille N, Sachais BS, Scales DC, Schwartz L, Shehata N, Turgeon AF, Wood H, Zarychanski R, Finzi A, Arnold DM, CONCOR-1 Study Group. 2021. Convalescent plasma for hospitalized patients with COVID-19: an open-label, randomized controlled trial. Nat Med 27:2012–2014. doi:10.1038/s41591-021-01488-2. 34504336 PMC8604729

[5] Abdelhady H, Abdelrazik M, Abdi Z, Abdo D, Abdulle A, Abel L, Abouzeenni S, Abrahamson G, Abusamra Y, Adams L, Adebambo O, Ademokun D, Adhikari N, Affron D, Aggarwal A, Agno R, Ahmad H, Ahmad N, Ahmed S, Ainscough K, Ainsworth J, Airoldi G, Aitken L, Ajeneye F, Akhtar N, Akinwumiju O, Al-Bayati M, Albert M, Alderman M, Alegria A, Alexander B, Alexander PD, Alfonso J, Ali H, Ali S, Allameddine A, Allan S, Allard I, Allen B, Allen B, Allen J, Allen L, Allen L, Allibone S, Ambrosino R, Amenyah K, Ammoun M, Anand R, Anand SP, Andersen K, et al. 2021. Effect of convalescent plasma on organ support-free days in critically ill patients with COVID-19: a randomized clinical trial. JAMA 326:1690–1702. doi:10.1001/jama.2021.18178. 34606578 PMC8491132

[6] Saeed S, O'Brien SF, Abe K, Yi Q-L, Rathod B, Wang J, Fazel-Zarandi M, Tuite A, Fisman D, Wood H, Colwill K, Gingras A-C, Drews SJ. 2021. Severe acute respiratory syndrome coronavirus 2 (SARS-CoV-2) seroprevalence: navigating the absence of a gold standard. PLoS One 16:e0257743. doi:10.1371/journal.pone.0257743. 34555095 PMC8459951

[7] Saeed S, Drews S, Pambrun C, Yi Q, Osmond L, O'Brien S. 2021. SARS-CoV-2 seroprevalence among blood donors after the first COVID-19 wave in Canada. Transfusion 61:862–872. doi:10.1111/trf.16296. 33527398 PMC8013879

[8] Saeed S, Drews SJ, Pambrun C, O'Brien SF. 2021. Waning anti-nucleocapsid IgG signal among SARS-CoV-2 seropositive blood donors: May–November 2020. JAMMI 6:10–11.36340216

[9] Drews SJ, Abe KT, Hu Q, Samson R, Gingras A-C, Colwill K, Rathod B, Wang J, Fazel-Zarandi M, Yi Q-L, Robinson A, Wood H, Tuite A, Fisman D, Evans DH, Lin Y-CJ, O'Brien SF. 2022. Resistance of SARS-CoV-2 beta and gamma variants to plasma collected from Canadian blood donors during the spring of 2020. Transfusion 62:37–43. doi:10.1111/trf.16713. 34662434 PMC8662190

[10] Drews SJ, Hu Q, Samson R, Abe KT, Rathod B, Colwill K, Gingras A-C, Yi Q-L, O'Brien SF. 2022. SARS-CoV-2 virus-like particle neutralizing capacity in blood donors depends on serological profile and donor-declared SARS-CoV-2 vaccination history. Microbiol Spectr 10:e0226221. doi:10.1128/spectrum.02262-21. 35171006 PMC8849073

[11] Abe K, Hu Q, Mozafarihashjin M, Samson R, Manguiat K, Robinson A, Rathod B, Hardy WR, Wang JH, Iskilova M, Pasculescu A, Fazel-Zarandi M, Li A, Paterson A, Chao G, Green K, Gilbert L, Barati S, Haq N, Takaoka A, Garnham Takaoka J, Quinn De, Launay K, Fahim C, Sheikh-Mohamed S, Arita Y, Durocher Y, Marcusson EG, Gommerman JL, Ostrowski M, Colwill K, Straus SE, Wood H, McGeer AJ, Gingras A-C. 2021. Neutralizing antibody responses to SARS-CoV-2 variants in vaccinated Ontario long-term care home residents and workers. medRxiv. doi:10.1101/2021.08.06.21261721.

[12] Colwill K, Galipeau Y, Stuible M, Gervais C, Arnold C, Rathod B, Abe KT, Wang JH, Pasculescu A, Maltseva M, Rocheleau L, Pelchat M, Fazel-Zarandi M, Iskilova M, Barrios-Rodiles M, Bennett L, Yau K, Cholette F, Mesa C, Li AX, Paterson A, Hladunewich MA, Goodwin PJ, Wrana JL, Drews SJ, Mubareka S, McGeer AJ, Kim J, Langlois M-A, Gingras A-C, Durocher Y. 2022. A scalable serology solution for profiling humoral immune responses to SARS-CoV-2 infection and vaccination. Clin Transl Immunology 11:e1380. doi:10.1002/cti2.1380. 35356067 PMC8942165

[13] Yau K, Chan CT, Abe KT, Jiang Y, Atiquzzaman M, Mullin SI, Shadowitz E, Liu L, Kostadinovic E, Sukovic T, Gonzalez A, McGrath-Chong ME, Oliver MJ, Perl J, Leis JA, Bolotin S, Tran V, Levin A, Blake PG, Colwill K, Gingras A-C, Hladunewich MA. 2022. Differences in mRNA-1273 (Moderna) and BNT162b2 (Pfizer-BioNTech) SARS-CoV-2 vaccine immunogenicity among patients undergoing dialysis. CMAJ 194:E297–E305. doi:10.1503/cmaj.211881. 35115375 PMC9053976

[14] Yau K, Abe KT, Naimark D, Oliver MJ, Perl J, Leis JA, Bolotin S, Tran V, Mullin SI, Shadowitz E, Gonzalez A, Sukovic T, Garnham-Takaoka J, de Launay KQ, Takaoka A, Straus SE, McGeer AJ, Chan CT, Colwill K, Gingras A-C, Hladunewich MA. 2021. Evaluation of the SARS-CoV-2 antibody response to the BNT162b2 vaccine in patients undergoing hemodialysis. JAMA Netw Open 4:e2123622. doi:10.1001/jamanetworkopen.2021.23622. 34473256 PMC8414193

[15] Liu J, Budylowski P, Samson R, Griffin BD, Babuadze G, Rathod B, Colwill K, Abioye JA, Schwartz JA, Law R, Yip L, Ahn SK, Chau S, Naghibosadat M, Arita Y, Hu Q, Yue FY, Banerjee A, Hardy WR, Mossman K, Mubareka S, Kozak RA, Pollanen MS, Martin Orozco N, Gingras AC, Marcusson EG, Ostrowski MA. 2021. Preclinical evaluation of a SARS-CoV-2 mRNA vaccine PTX-COVID19-B. Sci Adv 8:eabj9815. doi:10.1126/sciadv.abj9815. PMC8769538 35044832

[16] Cholette F, Mesa C, Harris A, Ellis H, Cachero K, Lacap P, Galipeau Y, Langlois M-A, Gingras A-C, Yansouni CP, Papenburg J, Cheng MP, Chakraborty P, Stein DR, Van Caeseele P, Bartlett S, Krajden M, Goldfarb D, McGeer A, Osiowy C, Hankins C, Mazer B, Drebot M, Kim J, COVID-19 Immunity Task Force (CITF) Working Group. 2021. Dried blood spot specimens for SARS-CoV-2 antibody testing: a multi-site, multi-assay comparison. PLoS One 16:e0261003. doi:10.1371/journal.pone.0261003. 34874948 PMC8651133

[17] Statistics Canada. 2021. Population estimates, quarterly. https://www150.statcan.gc.ca/t1/tbl1/en/tv.action?pid=1710000901. Retrieved 10 May 2022.

[18] Tang X, Sharma A, Pasic M, Brown P, Colwill K, Gelband H, Birnboim HC, Nagelkerke N, Bogoch II, Bansal A, Newcombe L, Slater J, Rodriguez PS, Huang G, Fu SH, Meh C, Wu DC, Kaul R, Langlois M-A, Morawski E, Hollander A, Eliopoulos D, Aloi B, Lam T, Abe KT, Rathod B, Fazel-Zarandi M, Wang J, Iskilova M, Pasculescu A, Caldwell L, Barrios-Rodiles M, Mohammed-Ali Z, Vas N, Santhanam DR, Cho ER, Qu K, Jha S, Jha V, Suraweera W, Malhotra V, Mastali K, Wen R, Sinha S, Reid A, Gingras A-C, Chakraborty P, Slutsky AS, Jha P, Ab-C Study Investigators. 2022. Assessment of SARS-CoV-2 seropositivity during the first and second viral waves in 2020 and 2021 among Canadian adults. JAMA Netw Open 5:e2146798. doi:10.1001/jamanetworkopen.2021.46798. 35171263 PMC8851304

[19] Walmsley S, Szadkowski L, Wouters B, Clarke R, Colwill K, Rochon P, Brudno M, Ravindran R, Raboud J, McGeer A, Oza A, Graham C, Silva A, Manase D, Parente L, Simpson J, Dayam RM, Pasculescu A, Gingras A-C. 2022. Safety and efficacy of preventative COVID vaccines: the StopCoV study. medRxiv. doi:10.1101/2022.02.09.22270734. PMC10312218 37397827

[20] Dayam RM, Law JC, Goetgebuer RL, Chao GYC, Abe KT, Sutton M, Finkelstein N, Stempak JM, Pereira D, Croitoru D, Acheampong L, Rizwan S, Rymaszewski K, Milgrom R, Ganatra D, Batista NV, Girard M, Lau I, Law R, Cheung MW, Rathod B, Kitaygorodsky J, Samson R, Hu Q, Hardy WR, Haroon N, Inman RD, Piguet V, Chandran V, Silverberg MS, Gingras A-C, Watts TH. 2022. Accelerated waning of immunity to SARS-CoV-2 mRNA vaccines in patients with immune mediated inflammatory diseases. JCI Insight e159721. doi:10.1172/jci.insight.159721. 35471956 PMC9220925

[21] Stein DR, Osiowy C, Gretchen A, Thorlacius L, Fudge D, Lang A, Sekirov I, Morshed M, Levett PN, Tran V, Kus JV, Gubbay J, Mohan V, Charlton C, Kanji JN, Tipples G, Serhir B, Therrien C, Roger M, Jiao L, Zahariadis G, Needle R, Gilbert L, Desnoyers G, Garceau R, Bouhtiauy I, Longtin J, El-Gabalawy N, Dibernardo A, Lindsay LR, Drebot M, Canadian Public Health Laboratory Network (CPHLN) Serology Task Force. 2021. Evaluation of commercial SARS-CoV-2 serological assays in Canadian public health laboratories. Diagn Microbiol Infect Dis 101:115412. doi:10.1016/j.diagmicrobio.2021.115412. 34425450 PMC8377389

[22] Galipeau Y, Greig M, Liu G, Driedger M, Langlois MA. 2020. Humoral responses and serological assays in SARS-CoV-2 infections. Front Immunol 11:610688. doi:10.3389/fimmu.2020.610688. 33391281 PMC7775512

[23] Quach C, Deeks S. 2021. COVID-19 vaccination: why extend the interval between doses? JAMMI 6:73–78. doi:10.3138/jammi-2021-0323. 36341029 PMC9608698

[24] Government of Canada. 2021. Coronavirus disease (COVID-19): outbreak update. https://www.canada.ca/en/public-health/services/diseases/2019-novel-coronavirus-infection.html?utm_campaign=hc-sc-phm-21-22&utm_medium=sem&utm_source=ggl&utm_content=ad-text-en&utm_term=coronavirus%20update%20canada&adv=2122-0008&id_campaign=12663296824&id_source=125900518968&id_content=511624188952&gclid=Cj0KCQiA-eeMBhCpARIsAAZfxZDaNLkoO11qdPc3fXEVj_OsKpKvN82TwvcUQugL-YzSVe8rrLhYAxQaAndjEALw_wcB&gclsrc=aw.ds. Retrieved 10 May 2022.

[25] COVID-19 Immunity Task Force. 2021. Vaccine-induced seroprevalence hits highest level to date, yet early fourth wave hitting those most at-risk: Canadian Blood Services August report. https://www.covid19immunitytaskforce.ca/vaccine-induced-seroprevalence-hits-highest-level-to-date-yet-early-fourth-wave-hitting-those-most-at-risk-canadian-blood-services-august-report/. Retrieved 10 May 2022.

[26] Jones JM, Stone M, Sulaeman H, Fink RV, Dave H, Levy ME, Di Germanio C, Green V, Notari E, Saa P, Biggerstaff BJ, Strauss D, Kessler D, Vassallo R, Reik R, Rossmann S, Destree M, Nguyen K-A, Sayers M, Lough C, Bougie DW, Ritter M, Latoni G, Weales B, Sime S, Gorlin J, Brown NE, Gould CV, Berney K, Benoit TJ, Miller MJ, Freeman D, Kartik D, Fry AM, Azziz-Baumgartner E, Hall AJ, MacNeil A, Gundlapalli AV, Basavaraju SV, Gerber SI, Patton ME, Custer B, Williamson P, Simmons G, Thornburg NJ, Kleinman S, Stramer SL, Opsomer J, Busch MP. 2021. Estimated US infection- and vaccine-induced SARS-CoV-2 seroprevalence based on blood donations, July 2020-May 2021. JAMA 326:1400–1409. doi:10.1001/jama.2021.15161. 34473201 PMC8414359

[27] Abbott. 2020. SARS-CoV-2 IgG II Quant, Architect package insert. Abbott Laboratories, Chicago, IL.

[28] Soeorg H, Jõgi P, Naaber P, Ottas A, Toompere K, Lutsar I. 2022. Seroprevalence and levels of IgG antibodies after COVID-19 infection or vaccination. Infect Dis (Lond) 54:63–71. doi:10.1080/23744235.2021.1974540. 34520315 PMC8442755

[29] Chamkhi S, Dhaouadi T, Sfar I, Mokni S, Jebri A, Mansouri D, Ghedira S, Ben Jemia E, Ben Boujemaa S, Houissa M, Aouina H, Ben Abdallah T, Gorgi Y. 2022. Comparative study of six SARS-CoV-2 serology assays: diagnostic performance and antibody dynamics in a cohort of hospitalized patients for moderate to critical COVID-19. Int J Immunopathol Pharmacol 36:20587384211073232. doi:10.1177/20587384211073232. 35113728 PMC8819577

[30] Fedele G, Stefanelli P, Bella A, Fiore S, Pancheri S, Benedetti E, Fabiani C, Leone P, Vacca P, Schiavoni I, Neri A, Carannante A, Simmaco M, Santino I, Zuccali MG, Bizzarri G, Magnoni R, Benetollo PP, Brusaferro S, Rezza G, Ferro A. 2021. Anti-SARS-CoV-2 antibodies persistence after natural infection: a repeated serosurvey in Northern Italy. Ann Ist Super Sanita 57:265–271. doi:10.4415/ANN_21_04_01. 35076416

[31] Kislaya I, Gonçalves P, Gómez V, Gaio V, Roquette R, Barreto M, Sousa-Uva M, Torres AR, Santos J, Matos R, Manita C, Almeida Santos J, Soeiro S, de Sousa R, Costa I, Verdasca N, Guiomar R, Rodrigues AP, ISN2COVID-19 Group. 2022. SARS-CoV-2 seroprevalence in Portugal following the third epidemic wave: results of the second National Serological Survey (ISN2COVID-19). Infect Dis (Lond) 54:418–424. doi:10.1080/23744235.2021.2025421. 35023439

[32] Rose R, Neumann F, Grobe O, Lorentz T, Fickenscher H, Krumbholz A. 2022. Humoral immune response after different SARS-CoV-2 vaccination regimens. BMC Med 20:31. doi:10.1186/s12916-021-02231-x. 35057798 PMC8776512

[33] Perkmann T, Perkmann-Nagele N, Koller T, Mucher P, Radakovics A, Marculescu R, Wolzt M, Wagner OF, Binder CJ, Haslacher H. 2021. Anti-spike protein assays to determine SARS-CoV-2 antibody levels: a head-to-head comparison of five quantitative assays. Microbiol Spectr 9:e0024721. doi:10.1128/Spectrum.00247-21. 34190591 PMC8552734

[34] Haase M, Lesny P, Anderson M, Cloherty G, Stec M, Haase-Fielitz A, Haarhaus M, Santos-Araújo C, Veiga PM, Macario F. 2022. Humoral immunogenicity and tolerability of heterologous ChAd/BNT compared with homologous BNT/BNT and ChAd/ChAd SARS-CoV-2 vaccination in hemodialysis patients: a multicenter prospective observational study. J Nephrol 1–12. doi:10.1007/s40620-022-01247-7. 35084719 PMC8792133

[35] Barreiro P, Sanz JC, San Román J, Pérez-Abeledo M, Carretero M, Megías G, Viñuela-Prieto JM, Ramos B, Canora J, Martínez-Peromingo FJ, Barba R, Zapatero A, Candel FJ. 2022. A pilot study for the evaluation of an interferon-gamma release assay (IGRA) to measure T-cell immune responses after SARS-CoV-2 infection or vaccination in a unique cloistered cohort. J Clin Microbiol 60:e0219921. doi:10.1128/jcm.02199-21. 35020419 PMC8925901

[36] Kanji JN, Bailey A, Fenton J, Ling SH, Rivera R, Plitt S, Sligl WI, Taylor S, Turnbull LAnn, Tipples G, Charlton CL. 2021. Detection of SARS-CoV-2 antibodies formed in response to the BNT162b2 and mRNA-1237 mRNA vaccine by commercial antibody tests. Vaccine 39:5563–5570. doi:10.1016/j.vaccine.2021.08.022. 34454782 PMC8354789

[37] COVID-19 Immunity Task Force. 2021. Poorer neighbourhoods and racialized communities continue to lag behind in vaccine coverage: latest Canadian Blood Services results. https://www.covid19immunitytaskforce.ca/poorer-neighbourhoods-and-racialized-communities-continue-to-lag-behind-in-vaccine-coverage-latest-canadian-blood-services-results/. Retrieved 10 May 2022.

[38] Levin EG, Lustig Y, Cohen C, Fluss R, Indenbaum V, Amit S, Doolman R, Asraf K, Mendelson E, Ziv A, Rubin C, Freedman L, Kreiss Y, Regev-Yochay G. 2021. Waning immune humoral response to BNT162b2 COVID-19 vaccine over 6 Months. N Engl J Med 385:e84. doi:10.1056/NEJMoa2114583. 34614326 PMC8522797

[39] Kwok SL, Cheng SM, Leung JN, Leung K, Lee CK, Peiris JM, Wu JT. 2022. Waning antibody levels after COVID-19 vaccination with mRNA Comirnaty and inactivated CoronaVac vaccines in blood donors, Hong Kong, April 2020 to October 2021. Euro Surveill 27:2101197. doi:10.2807/1560-7917.ES.2022.27.2.2101197. 35027105 PMC8759113

[40] Krutikov M, Palmer T, Tut G, Fuller C, Azmi B, Giddings R, Shrotri M, Kaur N, Sylla P, Lancaster T, Irwin-Singer A, Hayward A, Moss P, Copas A, Shallcross L. 2022. Prevalence and duration of detectable SARS-CoV-2 nucleocapsid antibodies in staff and residents of long-term care facilities over the first year of the pandemic (VIVALDI study): prospective cohort study in England. Lancet Healthy Longev 3:e13–e21. doi:10.1016/S2666-7568(21)00282-8. 34935001 PMC8676418

[41] Søfteland JM, Gisslén M, Liljeqvist J‐Å, Friman V, de Coursey E, Karason K, Ekelund J, Felldin M, Magnusson J, Baid‐Agrawal S, Wallquist C, Schult A, Jacobsson H, Bergdahl A, Bemark M, Andersson L‐M, Holm Gunnarsson I, Stenström J, Leach S. 2022. Longevity of anti-spike and anti-nucleocapsid antibodies after COVID-19 in solid organ transplant recipients compared to immunocompetent controls. Am J Transplant 22:1245–1252. doi:10.1111/ajt.16909. 34860447 PMC9906230

[42] Nayak K, Gottimukkala K, Kumar S, Reddy ES, Edara VV, Kauffman R, Floyd K, Mantus G, Savargaonkar D, Goel PK, Arora S, Rahi M, Davis CW, Linderman S, Wrammert J, Suthar MS, Ahmed R, Sharma A, Murali-Krishna K, Chandele A. 2021. Characterization of neutralizing versus binding antibodies and memory B cells in COVID-19 recovered individuals from India. Virology 558:13–21. doi:10.1016/j.virol.2021.02.002. 33706207 PMC7934698

[43] Roozendaal R, Solforosi L, Stieh DJ, Serroyen J, Straetemans R, Dari A, Boulton M, Wegmann F, Rosendahl Huber SK, van der Lubbe JEM, Hendriks J, Le Gars M, Dekking L, Czapska-Casey DN, Guimera N, Janssen S, Tete S, Chandrashekar A, Mercado NB, Yu J, Koudstaal W, Perez-Ruixo JJ, Sadoff J, Barouch DH, Schuitemaker H, Zahn R. 2021. SARS-CoV-2 binding and neutralizing antibody levels after Ad26.COV2.S vaccination predict durable protection in rhesus macaques. Nat Commun 12:5877. doi:10.1038/s41467-021-26117-x. 34620860 PMC8497464

[44] Niu L, Wittrock KN, Clabaugh GC, Srivastava V, Cho MW. 2021. A structural landscape of neutralizing antibodies against SARS-CoV-2 receptor binding domain. Front Immunol 12:647934. doi:10.3389/fimmu.2021.647934. 33995366 PMC8113771

[45] Yu Y, Wang M, Zhang X, Li S, Lu Q, Zeng H, Hou H, Li H, Zhang M, Jiang F, Wu J, Ding R, Zhou Z, Liu M, Si W, Zhu T, Li H, Ma J, Gu Y, She G, Li X, Zhang Y, Peng K, Huang W, Liu W, Wang Y. 2021. Antibody-dependent cellular cytotoxicity response to SARS-CoV-2 in COVID-19 patients. Signal Transduct Target Ther 6:346. doi:10.1038/s41392-021-00759-1. 34561414 PMC8463587

[46] Dufloo J, Grzelak L, Staropoli I, Madec Y, Tondeur L, Anna F, Pelleau S, Wiedemann A, Planchais C, Buchrieser J, Robinot R, Ungeheuer M-N, Mouquet H, Charneau P, White M, Lévy Y, Hoen B, Fontanet A, Schwartz O, Bruel T. 2021. Asymptomatic and symptomatic SARS-CoV-2 infections elicit polyfunctional antibodies. Cell Rep Med 2:100275. doi:10.1016/j.xcrm.2021.100275. 33899033 PMC8057765

[47] Díez JM, Romero C, Cruz M, Vandeberg P, Merritt WK, Pradenas E, Trinité B, Blanco J, Clotet B, Willis T, Gajardo R. 2022. Anti-severe acute respiratory syndrome coronavirus 2 hyperimmune globulin demonstrates potent neutralization and antibody-dependent cellular cytotoxicity and phagocytosis through N and S proteins. J Infect Dis 225:938–946. doi:10.1093/infdis/jiab540. 34693968 PMC8574314

[48] Chen X, Rostad CA, Anderson LJ, Sun H-Y, Lapp SA, Stephens K, Hussaini L, Gibson T, Rouphael N, Anderson EJ. 2021. The development and kinetics of functional antibody-dependent cell-mediated cytotoxicity (ADCC) to SARS-CoV-2 spike protein. Virology 559:1–9. doi:10.1016/j.virol.2021.03.009. 33774551 PMC7975276

[49] Beaudoin-Bussières G, Chen Y, Ullah I, Prévost J, Tolbert WD, Symmes K, Ding S, Benlarbi M, Gong SY, Tauzin A, Gasser R, Chatterjee D, Vézina D, Goyette G, Richard J, Zhou F, Stamatatos L, McGuire AT, Charest H, Roger M, Pozharski E, Kumar P, Mothes W, Uchil PD, Pazgier M, Finzi A. 2022. A Fc-enhanced NTD-binding non-neutralizing antibody delays virus spread and synergizes with a nAb to protect mice from lethal SARS-CoV-2 infection. Cell Rep 38:110368. doi:10.1016/j.celrep.2022.110368. 35123652 PMC8786652

[50] COVID-19 Immunity Task Force. 2021. Recent blood donor data suggest that Canadians still remain vulnerable to SARS-CoV-2 infection. https://www.covid19immunitytaskforce.ca/recent-blood-donor-data-suggest-that-canadians-still-remain-vulnerable-to-sars-cov-2-infection/. Retrieved 10 May 2022.

[51] Fenioux C, Teixeira L, Fourati S, Melica G, Lelievre JD, Gallien S, Zalcman G, Pawlotsky JM, Tournigand C. 2022. SARS-CoV-2 antibody response to 2 or 3 doses of the BNT162b2 vaccine in patients treated with anticancer agents. JAMA Oncol 8:612–617. doi:10.1001/jamaoncol.2021.7777. 34994776 PMC8742219

[52] Swadźba J, Anyszek T, Panek A, Martin E. 2021. Anti-spike SARS-CoV-2 IgG assessment with a commercial assay during a 4-month course after COVID-19 vaccination. Vaccines 9:1367. doi:10.3390/vaccines9111367. 34835298 PMC8617658

[53] National Collaborating Centre for Infectious Diseases. 2021. Updates on COVID-19 variants of concern. https://nccid.ca/covid-19-variants/#subMenuSection0. Retrieved 10 May 2022.

[54] Canadian Blood Services. 2019. Surveillance report. https://professionaleducation.blood.ca/en/transfusion/publications/surveillance-report. Retrieved 10 May 2022.

[55] Landis JR, Koch GG. 1977. The measurement of observer agreement for categorical data. Biometrics 33:159–174. doi:10.2307/2529310. 843571

[56] GraphPad. 2022. QuickCalcs. https://www.graphpad.com/quickcalcs/. Retrieved 10 May 2022.

[57] Lowry R. 2021. Vassar Stats clinical calculator 1. http://vassarstats.net/clin1.html. Retrieved 10 May 2022.

[58] Cohen JA. 1960. A coefficient of agreement for nominal scales. Educ Psychol Meas 20:37–20. doi:10.1177/001316446002000104.

